# An evaluation of the rat intestinal monoamine biogeography days following exposure to acute stress

**DOI:** 10.3389/fphys.2022.1021985

**Published:** 2022-12-13

**Authors:** Ella E. Bauer, Carter H. Reed, Mark Lyte, Peter J. Clark

**Affiliations:** ^1^ Department of Food Science and Human Nutrition, Iowa State University, Ames, IA, United States; ^2^ Department of Kinesiology, Iowa State University, Ames, IA, United States; ^3^ Department of Veterinary Microbiology and Preventive Medicine, Iowa State University, Ames, IA, United States

**Keywords:** acute stress, intestinal monoamines, gut dopamine, gut serotonin, intestinal biogeography, functional gastrointestinal disease, gastrointestinal distress

## Abstract

Stress-induced abnormalities in gut monoamine levels (e.g., serotonin, dopamine, norepinephrine) have been linked to gastrointestinal (GI) dysfunction, as well as the worsening of symptoms in GI disorders. However, the influence of stress on changes across the entire intestinal monoamine biogeography has not been well-characterized, especially in the days following stress exposure. Therefore, the aim of this study was to comprehensively assess changes to monoamine neurochemical signatures across the entire rat intestinal tract days after exposure to an acute stressor. To the end, adult male F344 rats were subjected to an episode of unpredictable tail shocks (acute stress) or left undisturbed. Forty-eight hours later rats were euthanized either following a 12 h period of fasting or 30 min of food access to evaluate neurochemical profiles during the peri- and early postprandial periods. Monoamine-related neurochemicals were measured *via* UHPLC in regions of the small intestine (duodenum, jejunum, ileum), large intestine (cecum, proximal colon, distal colon), cecal contents, fecal contents, and liver. The results suggest a relatively wide-spread increase in measures of serotonin activity across intestinal regions can be observed 48 h after exposure to acute stress, however some evidence was found supporting localized differences in serotonin metabolization. Moreover, acute stress exposure reduced catecholamine-related neurochemical concentrations most notably in the ileum, and to a lesser extent in the cecal contents. Next, stress-related fecal serotonin concentrations were consistent with intestinal profiles. However, fecal dopamine was elevated in association with stress, which did not parallel findings in any other intestinal area. Finally, stress exposure and the food access period together only had minor effects on intestinal monoamine profiles. Taken together, these data suggest nuanced differences in monoaminergic profiles exist across intestinal regions the days following exposure to an acute stressor, highlighting the importance of assessments that consider the entire intestinal tract biogeography when investigating stress-related biological outcomes that may be relevant to GI pathophysiology.

## 1 Introduction

Exposure to adverse experiences (e.g., psychological stress) is not only a major risk factor for gastrointestinal (GI) dysfunction, but can also worsen functional gastrointestinal disorders (FGIDs) symptomology (Garrick et al., 1989; Morrow and Garrick, 1997; Mayer, 2000; Konturek et al., 2011). Indeed, psychological stressors can alter GI functions, including motility, secretions, visceral sensitivity, and mucosal barrier integrity (Konturek et al., 2011). Moreover, a high comorbidity exists between the development of FDIGs and posttraumatic stress disorder (PTSD) (Bradford et al., 2012; Pohl et al., 2015; Park et al., 2016; Gradus et al., 2017; Noejovich et al., 2020). However, identifying and treating the underlying pathophysiology of stress-related GI dysfunction and FGIDs has proven to be a challenge (Wang and Camilleri, 2019), possibly due, at least in part, to the non-homogeneity of physiological environments and functions across the intestinal regions (Bowcutt et al., 2014; Donaldson et al., 2015; Kennedy and Chang, 2020; Bauer et al., 2021). Despite this, studies examining physiological responses to stress across the entire intestinal biogeography remain limited, outside of the microbiome. Thus, comprehensive multipoint investigations of the intestines could provide key insights into the region-specific physiological changes that develop following stress exposure, thereby providing important groundwork for targeted approaches that can better manage stress-related GI dysfunction.

Despite the clear link between stress and GI distress, significant gaps of knowledge exist concerning the physiological mechanisms by which stress exposure can disrupt GI functions. Converging lines of evidence suggest that monoamine neurochemicals (i.e., serotonin dopamine, norepinephrine) play central roles in regulating physiological functions of the GI tract (e.g., motility, secretions, permeability, blood flow, etc.) (Mittal et al., 2017). In fact, nearly 95% and 50% of the body’s serotonin (5-HT) and dopamine (DA), respectively, are produced in the gut, underscoring the importance of these neurochemicals to gut health (Bertaccini, 1960; Eisenhofer et al., 1997; Erspamer and Testini, 2011; Terry and Margolis, 2017). Therefore, it is not surprising that abnormalities in intestinal monoamine concentrations, such as sufficiently elevated 5-HT or lowered DA levels, can induce inflammatory GI conditions, thereby increasing intestinal motility and visceral sensitivity, which are key features of GI distress (Tonini et al., 2004; Bokic et al., 2015; Mittal et al., 2017). Given the link between stress and GI dysfunction (Söderholm and Perdue, 2001; Konturek et al., 2011; Ng et al., 2019), it is possible that stress-related abnormalities in intestinal monoamines may contribute to the development of GI distress and worsening of FGID symptoms. Indeed, some evidence exists suggesting pharmacological or probiotic approaches that can reduce the activity of intestinal 5-HT following stress exposure may be effective at attenuating rodent GI distress (Hirata et al., 2008; Li et al., 2019b). However, it is still unclear how stress influences the entire intestinal monoaminergic biogeography, as research has been typically limited to the examination of a single monoamine that is characterized within one or two gut regions, or just in fecal matter (Mönnikes et al., 2001; Dong et al., 2016, 2017; Li et al., 2019b; Hirabayashi et al., 2020; Li et al., 2022). This is an important consideration, as monoamine neurotransmitters interact in complex and coordinated manners to influence biological processes and sub-regions of the gut are not autonomous in function (Bowcutt et al., 2014; Donaldson et al., 2015; Kennedy and Chang, 2020; Bauer et al., 2021).

The purpose of this study was to investigate changes to monoamine-related neurochemical profiles across rat intestinal subregions that exist days after a single episode of acute stress. The unpredictable tail shock paradigm (i.e., acute stress) was chosen due to its comprehensively characterized stress physiology, likened to a relatively short-lived episode of PTSD (Miller and McEwen, 2006; Verbitsky et al., 2020), which has spanned over 5 decades of research (Weiss, 1968; Frank et al., 2013; Maier and Seligman, 2016; Fleshner and Crane, 2017; Baratta and Maier, 2019). Moreover, the unpredictable tail shock paradigm has been argued to be a promising model for understanding the biological underpinnings of GI dysfunction that are common with PTSD (Stam et al., 1997). Indeed, rats display several symptoms consistent with GI dysfunction following exposure to this stressor, including abnormal gastral contractility and mucosal injury (Weiss, 1968; Abbott et al., 1984; Garrick et al., 1989; Tsuda et al., 2013), alterations to the microbiome (Thompson et al., 2020), evidence of increased intestinal permeability (Maslanik et al., 2012), persistent hypophagia (Reed et al., 2021), as well as suppressed colonic contractility and increased fecal output that is watery in consistency (Morrow and Garrick, 1997). However, despite these observations, little work has been completed investigating the possible intestinal physiological underpinnings of GI distress using this model. Moreover, rats display exaggerated physiological and behavioral stress responses (Laudenslager et al., 1988; Fleshner et al., 1995; Maier and Watkins, 2005; Maier and Seligman, 2016) for days following exposure to this stressor in manners that may be relevant for understanding the development of more persistent GI distress symptoms (Greenwood-Van Meerveld and Johnson, 2017). However, to the best of our knowledge, no research has been completed characterizing stress-related monoaminergic responses beyond periods in close proximity to stress exposure (e.g., with a 1–2 h) (Mayer, 2000; Dong et al., 2017; Li et al., 2019b, 2022; Lyte et al., 2020).

To that end, rats were exposed an episode of unpredictable tail shocks and monoamine neurochemical profiles were assessed 48 h later in the small intestine (duodenum, jejunum, ileum) and large intestine (cecum, proximal colon, distal colon), as well as the cecal and fecal contents, and the liver. The concentrations of serotonin (5-HT), dopamine (DA), norepinephrine (NE), as well as the 5-HT metabolite 5-hydroxyindoleacetic acid (5-HIAA) and primary DA metabolites 3,4-dihydroxyphenylacetic acid (DOPAC) and homovanillic acid (HVA) were measured using Ultra-High-Performance Liquid Chromatography (uHPLC). Salsolinol (SAL), a bioactive metabolite of dopamine, was also measured because it has been implicated in gut dysfunction, but remains poorly characterized in response to environmental challenges (e.g., stress) (Kurnik-Łucka et al., 2018). This comprehensive multipoint assessment of the intestinal monoamine biogeography could have implications for intestine region-specific pathophysiology that develops after exposure to adverse experiences.

## 2 Materials and methods

### 2.1 Animals

Upon arrival, 225–250 g male Fischer 344 rats (*n* = 36) were housed 2-3 per cage. Male rats were used for this study as the vast majority of over five decades of research on stress physiology research has been completed in this exact paradigm using male Sprague Dawley or Fisher 344 rats (reviewed in Weiss, 1968; Frank et al., 2013; Christianson and Greenwood, 2013; Maier and Seligman, 2016; Fleshner and Crane, 2017; Baratta and Maier, 2019), which could provide behavioral or physiological context to any relevant intestinal outcomes. Rats were group housed to minimize the possible influence of chronic social isolation stress (Weiss et al., 2004) that could interact with the single episode of acute stress (described below), employed in this study, to confound the dependent measures. Group housed rats display heightened behavioral and physiological stress responses that have been likened to PTSD using the stress paradigm employed herein (for example, see Frank et al., 2007; Christianson et al., 2009; Christianson et al., 2010; Maslanik et al., 2012; Amat et al., 2014). No experimental manipulations took place during the first week while rats acclimated to the new vivarium. Rats in each cage were distinguished by marking with a different colored permanent marker on the base of the tails. Room temperature (21°C ± 1°C) and photoperiod (12:12 light/dark) were controlled for the entire study. There was an unexpected loss of one rat that was not included in analyses. All procedures were approved by the Iowa State University Institutional Animal Care and Use Committee (IACUC; Protocol #IACUC-18-298) and special care was taken to minimize animal discomfort.

### 2.2 Experimental design

**FIGURE 1:**
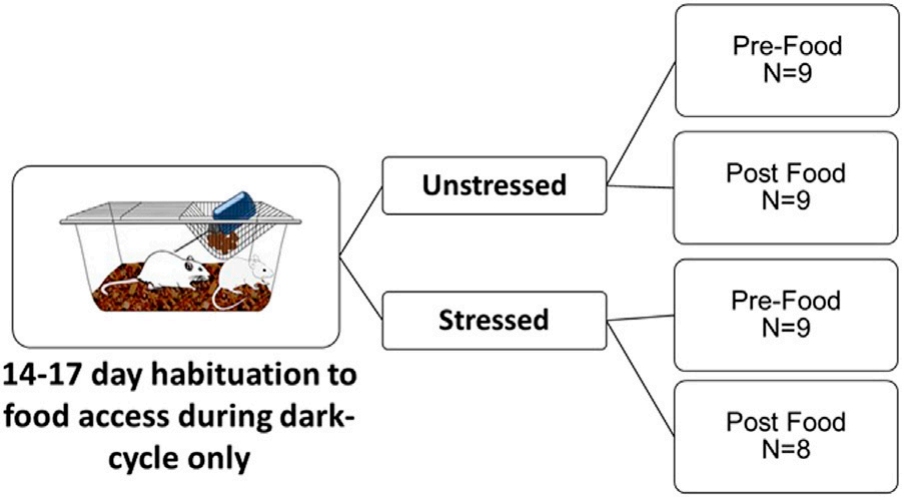
Experimental design. Adult male F344 rats were habituated to receive access to daily food only during the dark photoperiod over 14–17 days. Rats were then either left undisturbed in home cages (Unstressed) or exposed to an episode of unpredictable tail shocks (Stressed). Forty-eight hours after stress, rats were sampled either 15 min prior to food access (Pre-Food) or 30 min after food access (Post Food) to measure monoamine related neurochemicals across the intestines, intestinal contents, and liver using uHPLC.

Water was provided *ad libitum* throughout the experiment. After the first week, food access (ENVIGO Tekland 2014) was removed from cages during the light photoperiod, but provided *ad libitum* during the dark photoperiod (i.e., when rats are most active). This feeding schedule was completed so that intestinal measures could be obtained without food present (details below), to reduce the possible variation of dependent measures due to differences in chyme across regions of the intestines. Rats were acclimated to the 12 h of restricted food access for 14–17 days after which rats were exposed to a single episode of unpredictable tail shocks (Stressed group; described in detail below) or left undisturbed in their home cages (Unstressed group). Rats were exposed to stress in four cohorts (i.e., on either days 14, 15, 16, or 17, one cohort per day) so that post-euthanasia tissue collection could occur rapidly and at the same times across the groups (Figure 1).

All rats were euthanized by rapid decapitation without anesthesia 48 h after stress within two 15-min windows either before food access (Pre-Food group) or after 30 min of food access (Post-Food group). Forty-eight hours was chosen because it is sufficiently beyond stress exposure yet still within the approximately 72-h period that rat’s display heightened physiological responses to stress that have been compared to human PTSD (Stam et al., 1997; Hammack et al., 2003; Greenwood et al., 2012; Christianson and Greenwood, 2013; Frank et al., 2016). Rats in the Pre-Food group (*n* = 9 Unstressed, *n* = 9 Stressed) were sampled after a 12 h period fasting. The Pre-Food group was designed to observe intestinal neurochemical responses to stress without the presence of food in the GI tract, to minimize possible confounds in neurochemical data that could occur due to stress-induced reductions in eating (Reed et al., 2021). Rats in the Post-Food group (*n* = 9 Unstressed, *n* = 8 Stressed) were sampled 30 min after food access. The Post-Food condition was selected to model possible neurochemical changes that might contribute to the understanding of stress-related postprandial abdominal discomfort. In humans, postprandial abdominal discomfort commonly occurs in close approximation to eating (Steinsvik et al., 2020) (e.g., often within a couple of hours). The 30-min food access time point selected may be relevant to detect possible related monoamine involvement, due to the considerably faster GI transit process in rats compared to humans (Gao et al., 2017).

### 2.3 Acute stress paradigm

Cages (consisting of 2-3 rats) were randomly assigned to receive uncontrollable tail shocks (Stress) or remained in their home cages (Unstressed). The tail shock paradigm followed our previous publications (Clark et al., 2014, 2015; Reed et al., 2021). Briefly, the procedure took place in a room adjacent to the room they were housed. Rats were restrained using flat-bottom Plexiglas tubes with the tail exposed in the back approximately 2–5 h into the light cycle. Electrodes placed on the tail delivered 100 5-s tail shocks at variable 1-min intershock intervals operated by Med-PC software (Med Associates Inc.). The first 50 shocks were at 1.0 mA and the last 50 shocks were at 1.5 mA. Rats were returned to their home cages immediately following the tail shocks. The unpredictable tail shock paradigm is one of the most comprehensively studied models in stress physiology (Maier and Watkins, 2005; Maier and Seligman, 2016). As such, stress responses including, but not limited to, circulating corticosterone responses, glycemic levels, and sterile-systemic inflammation have been exhaustively characterized in past work (for example, see Frank et al., 2007; Maslanik et al., 2012; Beninson et al., 2014; Clark et al., 2014; Cox et al., 2014; Speaker et al., 2014; Conoscenti et al., 2019).

### 2.4 Neurochemical analysis

Small intestine regions (i.e., duodenum, jejunum, ileum), large intestine regions (i.e., cecum, proximal colon, distal colon), and the liver, as well as cecal and fecal contents were rapidly dissected in each rat on a glass plate placed over ice (Bauer et al., 2021). Intestinal tissues, intestinal contents, and liver samples were immediately placed into 0.2 M perchloric acid and flash frozen in liquid nitrogen and stored at −80°C until neurochemical analysis. Neurochemical detection was achieved using ultra-high-performance liquid chromatography. Detailed Ultra High Performance Liquid Chromatography methodology can be found in our previous publications (Bauer et al., 2021; Buhr et al., 2021; Reed et al., 2021). Neurochemical measurements for 5-HT, DA, and NE, as well as select metabolites (5-HIAA, DOPAC, HVA, SAL) were corrected for the weight of each sample (i.e., µg of neurochemical/gram of tissue) ± standard error of mean. The ratio of metabolite to neurotransmitter (5-HIAA/5-HT; DOPAC + HVA/DA) was calculated as a measure of neurotransmitter turnover (NJ et al., 1986; Peneder et al., 2011; Deurwaerdere et al., 2020; Bauer et al., 2021).

### 2.5 Statistical analysis

Group differences in neurochemical concentrations and turnover ratios were assessed using a Two-Way ANOVA for the factors acute stress (Unstressed or Stressed) and food access (Pre-Food or Post-Food). Statistically significant main effects between both stress exposure and food access, or an interaction between these factors were followed up with a Tukey corrected *post hoc* analysis. Due to the large volume of data acquired by analyzing several monoamine-related neurochemicals across multiple tissues sources, only statistically significant differences are reported in the results section. However, all neurochemical comparisons are located in the figures. Neurochemical concentration measurements that were greater than two standard deviations from the mean were excluded from the analysis, which is noted in the degrees of freedom. Also note, cage was considered a covariate, but did not influence the dependent measures. Therefore, the experimental unit for all analyses are at the level of individual rat (not cage). Statistical significance was set to 0.05 using SAS statistical software.

## 3 Results

### 3.1 Small intestine monoamines

#### 3.1.1 Duodenum

**FIGURE 2:**
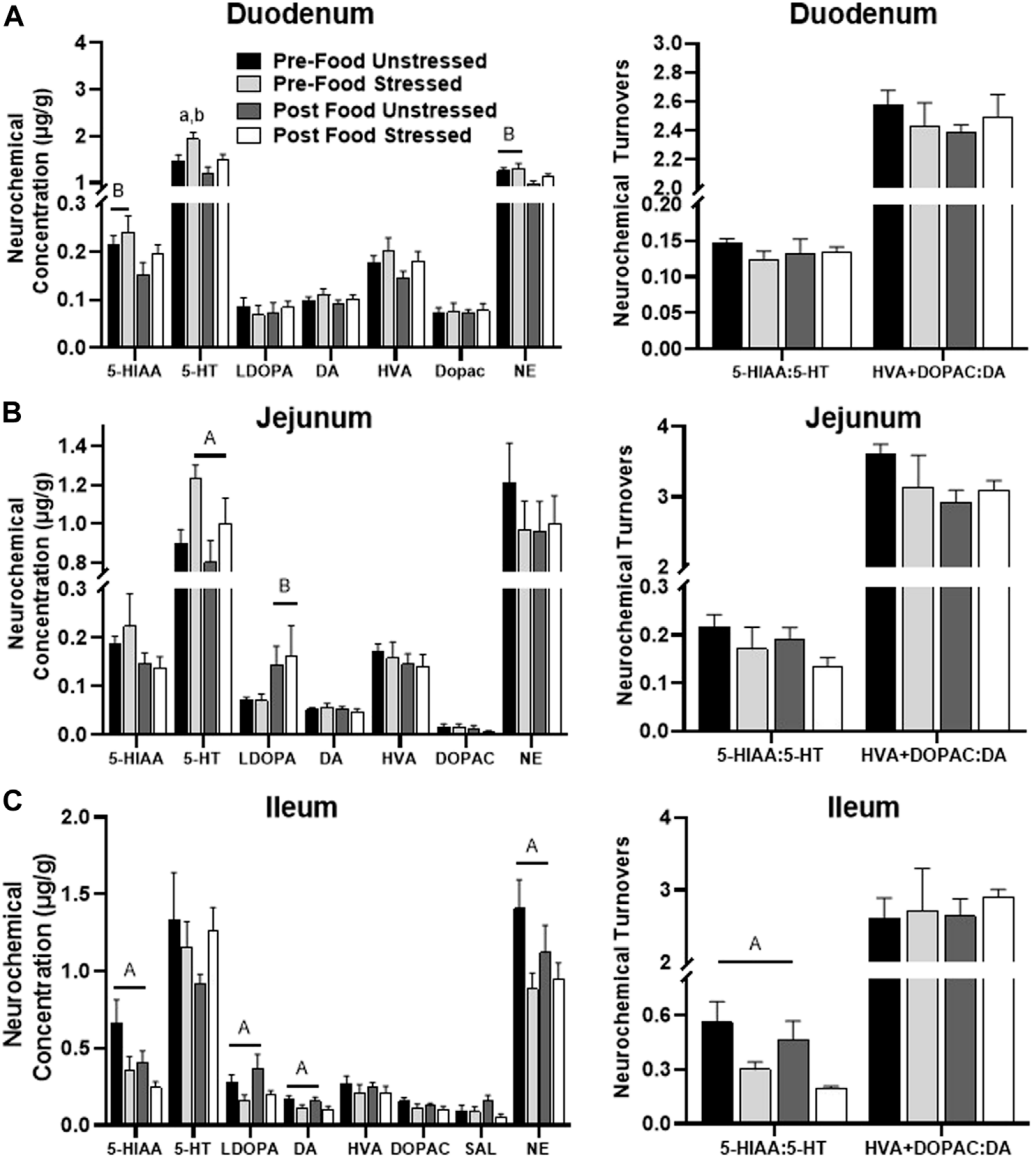
Monoamine-related neurochemical changes in the small intestines 48 h after stress exposure. **(A)** NE and stress-potentiated 5-HT concentrations were increased prior to food access in the duodenum. **(B)** Stress exposure increased 5-HT concentrations, whereas food access increased L-Dopa concentrations in the jejunum (left panel). **(C)** Stress lowered 5-HIAA, L-Dopa, DA, and NE concentrations (left panel), while also lowering 5-HT turnover measures (right panel) in the ileum.± S.E.M. Significant main effect of stress **(A)** or food access **(B)** from Two-Way ANOVA. **(A)** From Pre-Food Unstressed and **(B)** from Post Food Unstressed. Significance *p* < 0.05. Sample size *n* = 9 per group, except Post Food Stress group *n* = 8.

Stress exposure [*F*(1,31) = 7.66, *p =* 0.0095] and food access [*F*(1,31) = 7.40, *p =* 0.0106] influenced 5-HT concentrations. Post hoc analysis revealed that 5-HT was further elevated in Pre-Food Stress group compared to the Pre-Food (*p =* 0.0379) and Post Food Unstressed group (*p =* 0.0023) (Figure 2A). Additionally, the Post Food group had significantly lowered concentrations of the 5-HT metabolite 5-HIAA [*F*(1,31) = 4.33, *p =* 0.0459] and NE [*F*(1,31) = 7.81, *p =* 0.0089] compared to the Pre-Food group. SAL was below detectable limits.

#### 3.1.2 Jejunum

Stressed rats had significantly elevated 5-HT [*F*(1,31) = 7.36, *p =* 0.0108] compared to unstressed rats (Figure 2B). Additionally, rats in the Post Food group had significantly elevated concentrations of the DA precursor L-DOPA [*F*(1,31) = 5.40, *p =* 0.0269] compared to rats in the Pre-Food group. SAL was below detectable limits.

#### 3.1.3 Ileum

There were five stress-related neurochemical changes in the ileum. Stressed rats had decreased levels of 5-HIAA/5-HT [*F*(1,31) = 10.40, *p =* 0.0030], which was liked caused by lower 5-HIAA concentration [*F*(1,31) = 5.35, *p =* 0.0276] compared to unstressed rats (Figure 2C). Additionally, stress lowered concentrations of L-DOPA [*F*(1,31) = 6.30, *p =* 0.0175], DA [*F*(1,31) = 5.28, *p =* 0.0286], and NE [*F*(1,31) = 5.26, *p =* 0.0287] compared to unstressed rats. However, there were no significant neurochemical changes related to food access.

### 3.2 Large intestine, intestinal contents, and liver monoamines

#### 3.2.1 Cecum

**FIGURE 3:**
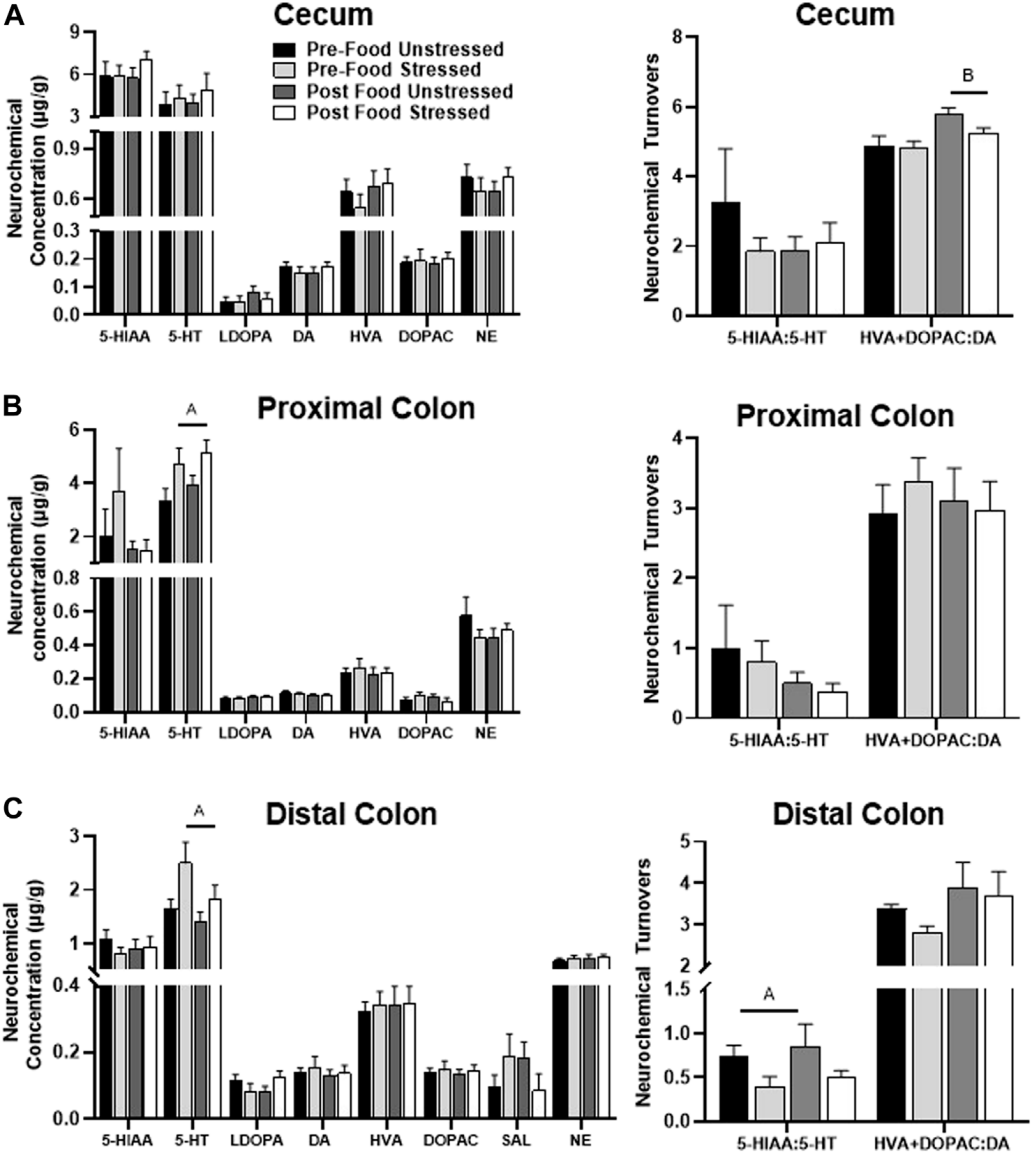
Monoamine-related neurochemical changes in the large intestines 48 h after stress exposure. **(A)** DA turnover measures were mildly elevated following food access (right panel), however no neurochemical responses to stress were observed in the cecum. **(B)** Stress exposure increased 5-HT concentrations (left panel) in the proximal colon. **(C)** Stress exposure increased 5-HT concentrations (left panel) with lower 5-HT turnover measures (right panel) in the distal colon. ±S.E.M. significant main effect of stress **(A)** or food access **(B)** from Two-Way ANOVA. Significance *p* < 0.05. Sample size *n* = 9 per group, except Post Food Stress group *n* = 8.

There were no significant stress-related neurochemical changes. However, rats in the Post Food group had elevated levels of DOPAC + HVA/DA [*F*(1,31) = 9.48, *p =* 0.0043] compared to rats in the Pre-Food group (Figure 3A). SAL was below detectable limits.

#### 3.2.2 Proximal colon

Stressed rats had elevated concentrations of 5-HT compared to unstressed rats [*F*(1,30) = 7.03 *p =* 0.0127] (Figure 3B). However, there were no significant neurochemical changes related to food access. SAL was below detectable limits.

#### 3.2.3 Distal colon

Stressed rats had lower levels of 5-HIAA/5-HT [*F*(1,31) = 4.21, *p =* 0.0487], which was likely due to an increase in 5-HT concentration [*F*(1,31) = 5.78, *p =* 0.0224], compared to unstressed rats (Figure 3C). Additionally, stress exposure and food access interacted to influence L-DOPA concentrations [*F*(1,31) = 4.22, *p =* 0.0485], however, *post hoc* analyses failed to reach significance.

#### 3.2.4 Cecal contents

**FIGURE 4:**
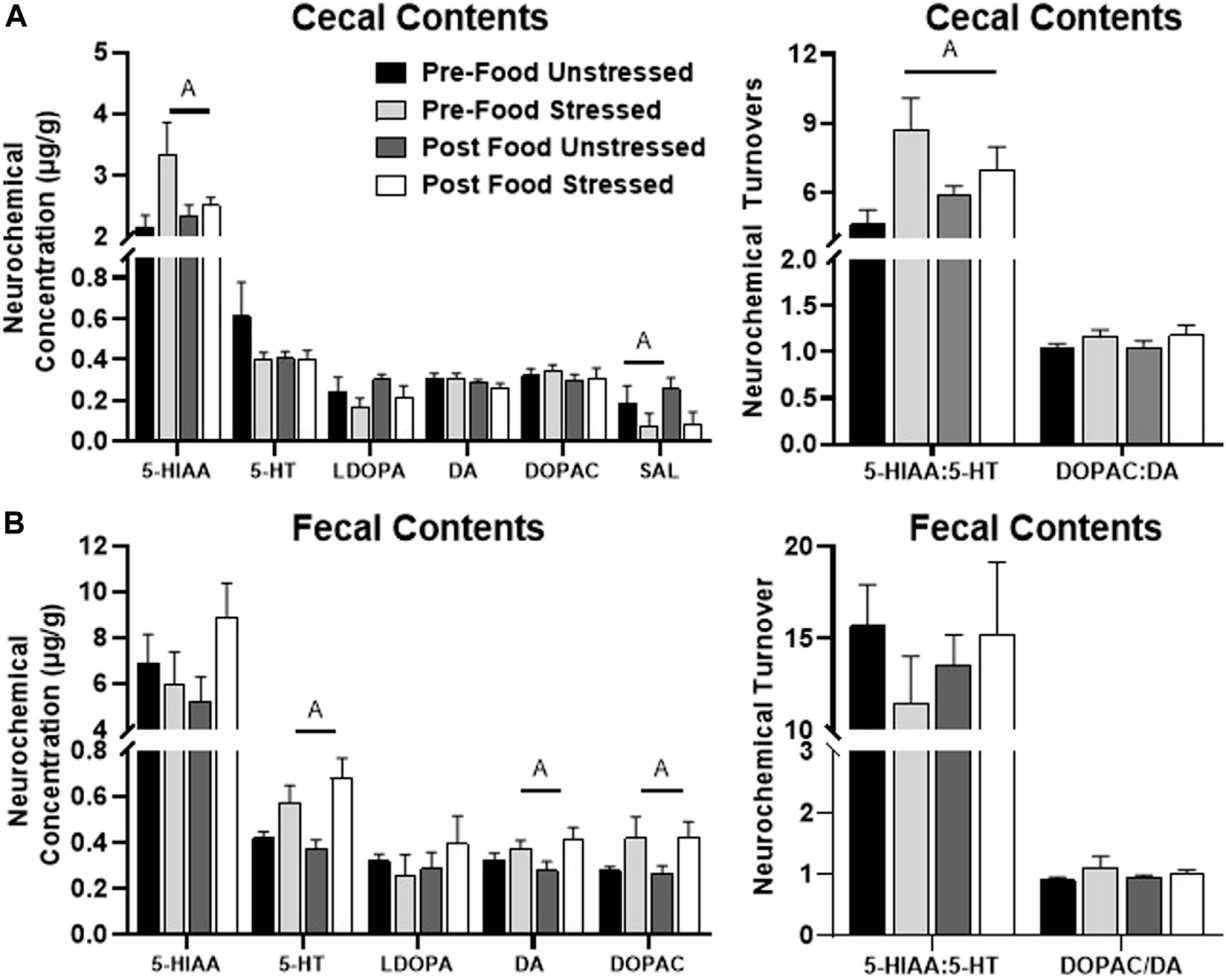
Monoamine-related neurochemical changes in the intestinal contents 48 h after stress exposure. **(A)** Stress increased 5-HIAA and decreased SAL concentrations (left panel), as well as elevated 5-HT turnover measures (right panel) in the cecal contents. **(B)** Stress increased 5-HT, DA, and DOPAC concentrations in the fecal contents (left panel). ±S.E.M. significant main effect of stress **(A)** or food access **(B)** from Two-Way ANOVA. Significance *p* < 0.05. Sample size *n* = 9 per group, except Post Food Stress group *n* = 8.

Stressed rats had elevated levels of 5-HIAA/5-HT [*F*(1,31) = 7.83, *p =* 0.0088], which was likely a result of increased 5-HIAA concentration [*F*(1,31) = 4.70, *p =* 0.0380] compared to unstressed rats (Figure 4A). Stress also lowered the concentration of SAL [*F*(1,31) = 4.67, *p =* 0.0385] compared to unstressed rats. However, there were no significant neurochemical changes related to food access. HVA was below detectable limits.

#### 3.2.5 Fecal contents

Stressed rats displayed higher concentrations of 5-HT [*F*(1,28) = 14.24, *p =* 0.0008], DA [*F*(28) = 5.07, *p =* 0.0324] and DOPAC [*F*(1,28) = 5.54, *p =* 0.0259] compared to unstressed rats (Figure 4B). There were no significant neurochemical changes related to food access. NE, HVA and SAL were below detectable limits.

#### 3.2.6 Liver

**FIGURE 5:**
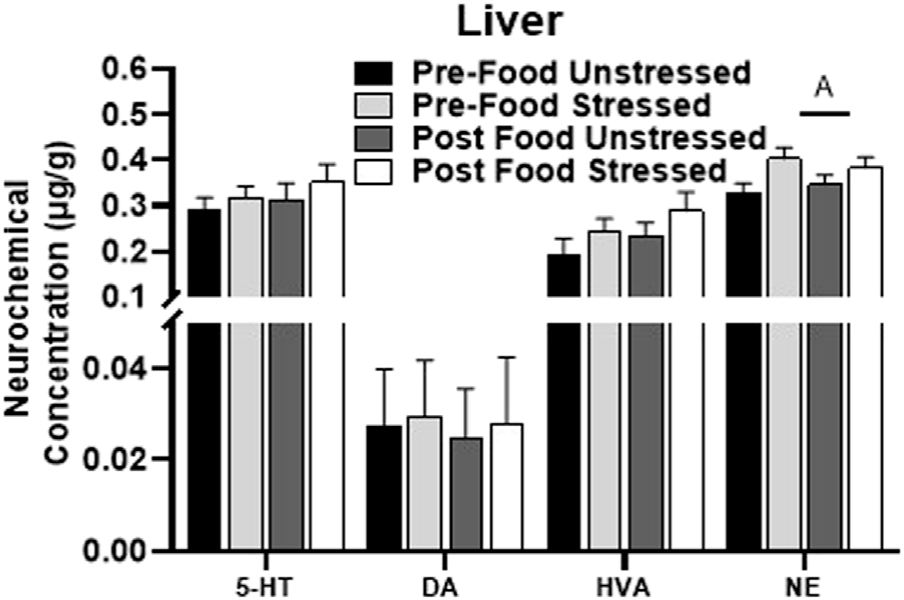
Monoamine-related neurochemical changes in the liver 48 h after stress exposure. Only a stress-induced increase NE concentrations was detected in the liver. ±S.E.M. significant main effect of stress (A) or food access (B) from Two-Way ANOVA. Significance *p* < 0.05. Sample size *n* = 9 per group, except Post Food Stress group *n* = 8.

Stressed rats had elevated levels of NE compared to unstressed rats in the liver [*F*(1,31) = 5.99, *p =* 0.0203] (Figure 5). There were no significant neurochemical changes related to food access. 5-HIAA, DOPAC, and SAL were below detectable limits, therefore 5-HIAA/5-HT and DOPAC + HVA/DA turnover ratios were not available. L-DOPA was not reliably detected across animals and was excluded from analysis.

## 4 Discussion

**FIGURE 6:**
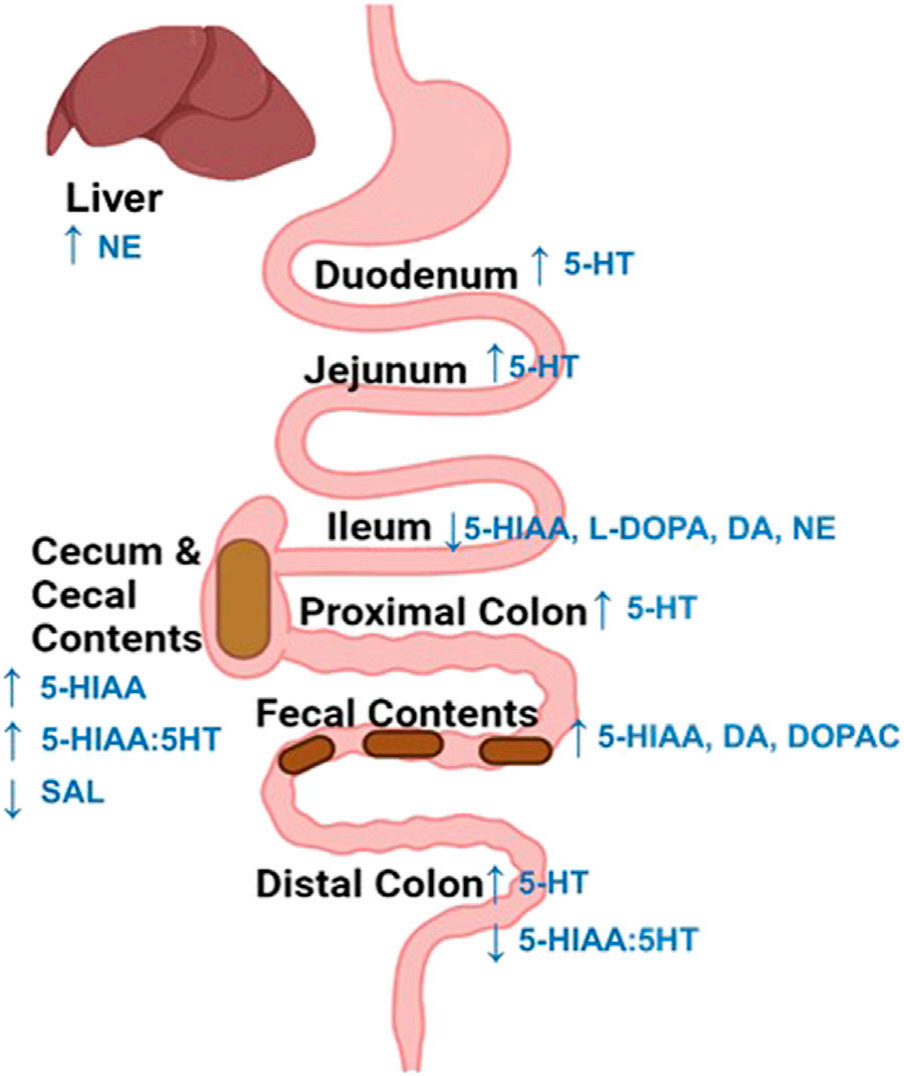
Summary of stress-induced changes to neurochemicals across the intestines and liver. An increase (up arrow) in 5-HT or its metabolite concentration were overserved across most intestinal regions 48 h following stress exposure. Whereas, catecholamine related neurochemicals were only notably altered in the Ileum, which displayed a reduction (down arrow) in L-DOPA, dopamine, and norepinephrine content. Finally, exposure to stress reduced (down arrow) salsolinol (SAL) concentrations in the cecal contents and increased norepinephrine concentrations in the liver.

The results of this study provide a relatively comprehensive assessment of changes to monoaminergic profiles across rat intestinal biogeography the days following exposure to an acutely adverse event. Several key findings were made that could have important implications for intestinal physiology and function during the days following acute stress. First, region-specific discrepancies in monoaminergic responses and metabolization were observed 48 h after acute stress exposure (Figure 6). These include evidence supporting intestinal region differences in the ability to metabolize stress-augmented 5-HT, as well as stress-induced reductions to catecholamine-related neurochemicals observed most notably in the ileum. These data could have implications for prevalence of stress-induced pathophysiological conditions within specific intestinal sub-regions. Second, stress-related neurochemical profiles in the fecal contents reflected a relatively widespread elevation in intestinal 5-HT concentrations. However, this was not the case for DA-related neurochemicals, where DA and metabolite concentrations were elevated in the feces but not across any intestinal areas. While surprising, the inconsistency of DA concentrations between the intestines and fecal matter could possibly be due to stress-induced increases of gastric-derived DA activity (not measured in this study). Nonetheless, these data highlight the importance of approaches that consider physiological changes across the entire GI tract, compared to a single intestinal area or just in fecal matter. Finally, access to food did not cause notable synergistic interactions with stress exposure to influence monoamine concentrations. Together, these findings shed light on localized changes to intestinal monoamine profiles that exist during the days after acute stress exposure, which may have important implications for more persistent risks of symptoms associated with GI distress (Greenwood-Van Meerveld and Johnson, 2017).

Despite the growing bodies of evidence supporting a role of abnormal monoamine activities in GI dysfunction, a comprehensive characterization of stress-induced changes to monoamines in the rodent intestinal tract is currently lacking, as studies predominately report only a single monoamine in one or two intestinal regions (typically the colon), or in fecal contents, within the first hour following stress exposure (Mayer, 2000; Dong et al., 2017; Li et al., 2019b; Li et al., 2022; Lyte et al., 2020). The extent of monoaminergic changes across each intestinal sub-region remains uncharacterized, especially in the days following exposure to an acute stressor. This is an important consideration, as monoamine neurotransmitters interact in complex and coordinated manners to influence intestinal processes, and sub-regions of the gut are not autonomous in function (Bowcutt et al., 2014; Donaldson et al., 2015; Kennedy and Chang, 2020; Bauer et al., 2021). In the current study, several region-specific changes to monoamine-related neurochemical concentrations were reported 48 h after stress exposure, which are consistent with inflammatory gut conditions that underlie the increased risk of GI distress (Tonini et al., 2004; Bokic et al., 2015; Mittal et al., 2017). For example, the cecal contents displayed stress-related increases in 5-HT turnover markers, without group differences in 5-HT levels, suggesting that this area may be more capable of metabolizing 5-HT in manners that could clear it more efficiently from local space. Whereas, duodenum and jejunum, as well as the proximal and distal colon displayed increases in 5-HT concentration, but not turnover, providing some evidence that these regions may not be as effective at metabolizing stress-augmented levels of 5-HT in intestinal space. Moreover, the ileum was the only intestinal region that displayed notable changes to catecholamine-related neurochemicals, whereby L-Dopa, DA, and NE concentrations were all reduced in this area following stress. Finally, the cecum appeared to be stress-robust to monoaminergic changes. These regional nuanced discrepancies in monoamine levels or metabolism during the days following stress exposure could be related, at least in part, to differences in bacterial composition that exists across regions of the intestine (Kennedy and Chang, 2020). For instance, gut microbiota have been shown to regulate 5-HT and DA levels in various manners, including, but not limited to, influencing host tryptophan metabolism, as well as 5-HT and DA metabolism (Yano et al., 2015; Moya-Pérez et al., 2017; Li et al., 2019b; Kaur et al., 2019; Kwon et al., 2019; Kennedy and Chang, 2020).

Yet still, stress-related differences of localized monoaminergic activities across intestinal regions may have implications for the etiology of GI distress symptoms, including those that have been reported in rats following immediately to days following exposure to unpredictable tail shocks (Weiss, 1970; Abbott et al., 1984; Garrick et al., 1989; Morrow and Garrick, 1997; Mika et al., 2017; Reed et al., 2021). Evidence indicates that sufficiently elevated 5-HT and lowered DA activity in the gut can promote inflammatory responses, thereby increasing intestinal motility and heightening visceral sensitivity (Galvin, 1992; Mawe et al., 2013; Galligan et al., 2013; Dong et al., 2017; Kwon et al., 2019; Alvarado and Ciroba., 2020; Steinsvik et al., 2020; Kurnik-Łucka et al., 2021). Therefore, region-specific differences in the capacity of the intestines to metabolize 5-HT or produce DA following stress may have localized consequences on intestinal health, which could contribute in distinct ways to features of GI dysfunctions following adverse experiences. For example, a stress-related augmentation of 5-HT concentrations along with the reduced turnover unique to the distal colon are consistent with a particularly prolonged exposure to heightened 5HT that could underlie the severity of pathology reported in this region. Indeed, chronic inflammation and ulcers are reported specifically within this region of people suffering from stress-related FDIGs (Alvarado and Ciorba, 2020; Pergolizzi et al., 2022). Taken together, these results suggest that nuances across intestinal sub-areas may exist in monoaminergic responses even days following exposure to a stressor of sufficient intensity. A further understanding of the biological factors contributing to region-specific monoaminergic stress responses could be topics for future investigations, as it may hold importance for understanding the localized intestinal conditions that contribute to specific symptoms of GI distress.

Additionally, it is worth noting the DA metabolite salsolinol (SAL) was also lowered by stress in the cecal contents. This is interesting because SAL has bioactive properties in the gut and brain (Banach et al., 2005; Zurowski et al., 2006; Gil et al., 2011; Kurnik-Łucka et al., 2018, 2020), yet remains poorly characterized following environmental challenges (e.g., stress). Some evidence suggests that a small range of SAL concentrations may be neuroprotective, however deviation from this range may become neurotoxic (Kurnik-Łucka et al., 2020). Therefore, sufficiently lower or higher levels of SAL could be detrimental to the enteric nervous system (Kurnik-Łucka et al., 2017). Indeed, abnormally elevated SAL levels in the gut have been implicated in increased immune and neural cell apoptosis, thereby contributing to diminished intrinsic and extrinsic mediated motility (Banach et al., 2005; Zurowski et al., 2006; Gil et al., 2011; Kurnik-Łucka et al., 2017). However, the effects of reduced SAL levels in the gut remains less characterized. Therefore, stress-related changes to intestinal SAL levels may have implications for GI health, which warrants further investigations. Indeed, very little is known about this bioactive DA metabolite, despite a growing literature linking it with both GI and neurodegenerative disorders (Antkiewicz-Michaluk et al., 1997; Qualls et al., 2014; Gonzalez et al., 2021).

Fecal biomarkers are commonly used as gross measures of GI mucosal physiology (Burri and Beglinger, 2014; Chong et al., 2019). For instance, fecal neurochemical concentrations commonly serve as a correlate of neurochemical patterns in intestinal mucosa environment (Assche, 2011; Hirabayashi et al., 2020a). Therefore, it is of interest to note the discrepancies between the patterns of 5-HT and DA levels observed in fecal content compared to the intestinal tissue, suggesting fecal neurochemical changes may not always reflect those across the entire intestinal tract. In the current study, fecal levels of 5-HT were consistent with the evidence supporting a relatively widespread increase of stress-related intestinal 5-HT activity. Whereas, the stress-elevated fecal DA concentrations did not parallel neurochemical observations across any intestinal area, whereby the few notable changes were primarily indicative of decreases, not increases, of DA content. One possibility is that elevated fecal DA levels may be reflective of stress-induced changes to gastric-derived DA (not assessed in this study). Previous work using pharmacological approaches suggests that stress alters gastric dopamine production, thus gastric-derived DA released into the luminal contents may reach distal segments of the intestinal tract possibly through the transit of chyme and fecal matter (Glavin, 1992; Mezey et al., 1996; Rasheed and Alghasham, 2012). However, the short and long-term effects of adverse experiences on gastric DA concentrations remain not well characterized. Taken together, these data suggest inconsistencies in patterns of detection for stress-induced neurochemicals may exist between the fecal contents and intestinal tissues. Future work should follow up on possible sources of elevated DA content in the fecal content that did not reflect intestinal DA concentration patterns, as a better understanding of sources contributing fecal neurochemical concentration changes could possibly facilitate the diagnosis and treatment of FGIDs.

Postprandial abdominal discomfort is a symptom of many GI disorders that often occurs in close proximity to eating (Steinsvik et al., 2020) and may be related, in part, to abnormal monoamine activity-mediated visceroperception. For example, abnormally elevated postprandial 5-HT is implicated in visceral hypersensitivity (Crowell, 2004; Spiller, 2007; Camilleri, 2009). Although the reason for abnormal postprandial 5-HT in GI disorders is not entirely clear, it is possible that following exposure to stressors of sufficient intensity, postprandial and stress-related 5-HT may synergistically interact to produce greater visceral discomfort in close approximation to eating (Steinsvik et al., 2020). However, only one additive 5-HT-related effect was observed in our study, which was for stressed rats prior to food access, not after food access, in the duodenum. One possible explanation for the lack of synergistic stress and food effects on monoamines could be that the stressed animals in this paradigm persistently eat less (Reed et al., 2021), which may be related to food avoidance from already present discomfort (Li et al., 2019a). Alternatively, it seems plausible that more monoaminergic changes would have been present if a time point was examined further from food access, as a food bolus can take about 12 h to traverse the extent of the intestines (Dalziel et al., 2016). The latter is consistent with the observation that the few food-related neurochemical effects present in this study were observed in the proximal intestinal regions. Thus, while perhaps less relevant for understanding the conditions that may contribute to stress-induced early postprandial abdominal discomfort, a longer duration of access to food prior to sampling may be necessary to observe synergistic effects between intestinal neurochemicals and food in the GI tract.

Some careful considerations should be given to interpretations in the current study. First, this study was completed in male rats. Some evidence suggests differences may exist between male and female intestinal monoaminergic systems (Viramontes et al., 2001; López-Contreras et al., 2008; Lyte et al., 2020; Bauer et al., 2021), which might contribute to reported variation in the risk of developing GI disorders between sexes. Second, the chosen methodology does not allow for the distinction between possible intestinal sublayer differences in monoamine changes (e.g., mucosa, submucosa, muscular, etc.) or specific cell types/sources (e.g., enterocytes, nerves, microbes, etc.) contributing to stress-related changes in monoamine levels. These types of analyses could be topics for future studies using highly targeted approaches that can obtain neurochemical measures at cellular levels. Finally, the current study did not establish a direct link between neurochemical outcomes and intestinal health. Several studies have characterized symptoms of GI dysfunction following exposure to this stress model, including abnormal gastral contractility and mucosal injury (Weiss, 1968; Abbott et al., 1984; Garrick et al., 1989; Tsuda et al., 2013), alterations to the microbiome (Thompson et al., 2020), evidence of increased intestinal permeability (Maslanik et al., 2012), persistent hypophagia (Reed et al., 2021), as well as suppressed colonic contractility and increased fecal output that is watery in consistency (Morrow and Garrick, 1997). Some of the stress-induced changes, like reductions in food consumption, persist for weeks in F344 rats (Reed et al., 2021). However, the physiological underpinnings and persistence of many of these intestinal outcomes remains uncharacterized in this model, which could have relevance for understanding GI health with PTSD (Stam et al., 1997; Conoscenti and Fanselow, 2019; Glynn et al., 2021). Future studies are needed to understand the exact contributions of region specific stress-induced changes to monoamine neurochemicals on possible long lasting disturbances to GI health.

In conclusion, the current study provides evidence supporting differences in the patterns of monoamine neurochemical profiles across the intestines during the days following exposure to an acute stressor. The region-specific profiles of stress-induced monoaminergic changes are consistent with pathophysiological states that may contribute to an increased risk for GI dysfunction, which have also been reported in rats following this stress paradigm, including some of that may last for days (Abbott et al., 1984; Garrick et al., 1989; Morrow and Garrick, 1997; Hirata et al., 2008; Maslanik et al., 2012; Tsuda et al., 2013; Li et al., 2019b; Thompson et al., 2020). Furthermore, the unpredictable tail shock paradigm may hold promise as a behavioral assay used alone or with existing rodent models of FDIGs to better understand more persistent forms of stress-induced changes to intestinal physiology, which may be particularly relevant for understanding the biological conditions underlying high comorbidity of GI disorders in those with PTSD (Stam et al., 1997; Conoscenti and Fanselow, 2019; Glynn et al., 2021). Future studies incorporating comprehensive, multipoint measures of physiological responses across the intestinal biogeography, such as in the present study, are an important step towards the development of highly targeted interventions that more effectively alleviate localized intestinal conditions that underlie the increased risk for developing GI distress.

## Data Availability

The raw data supporting the conclusion of this article will be made available by the authors, without undue reservation.

## Abbreviations

DA, dopamine; DOPAC, 3,4-dihydroxyphenylacetic acid; FGIDs, functional gastrointestinal disorders; GI, gastrointestinal; HVA, homovanillic acid; NE, norepinephrine; PTSD, post-traumatic stress disorder; SAL, salsolinol; UHPLC, ultra-high-performance liquid chromatography; 5-HT, serotonin; 5-HIAA, 5-hydroxyindoleacetic acid.
